# Correlation between Google Trends on dengue fever and national surveillance report in Indonesia

**DOI:** 10.1080/16549716.2018.1552652

**Published:** 2019-01-08

**Authors:** Atina Husnayain, Anis Fuad, Lutfan Lazuardi

**Affiliations:** a E-Health Division, Center for Health Policy and Management, Faculty of Medicine, Public Health and Nursing, Universitas Gadjah Mada, Yogyakarta, Indonesia; b Department of Biostatistics, Epidemiology, and Population Health, Faculty of Medicine, Public Health and Nursing, Universitas Gadjah Mada, Yogyakarta, Indonesia; c Department of Health Policy Management, Faculty of Medicine, Public Health and Nursing, Universitas Gadjah Mada, Yogyakarta, Indonesia

**Keywords:** Google Trends, information seeking, digital epidemiology, dengue, Indonesia

## Abstract

**Background**: Digital traces are rapidly used for health monitoring purposes in recent years. This approach is growing as the consequence of increased use of mobile phone, Internet, and machine learning. Many studies reported the use of Google Trends data as a potential data source to assist traditional surveillance systems. The rise of Internet penetration (54.7%) and the huge utilization of Google (98%) indicate the potential use of Google Trends in Indonesia. No study was performed to measure the correlation between country wide official dengue reports and Google Trends data in Indonesia.

**Objective**: This study aims to measure the correlation between Google Trends data on dengue fever and the Indonesian national surveillance report.

**Methods**: This research was a quantitative study using time series data (2012–2016). Two sets of data were analyzed using Moving Average analysis in Microsoft Excel. Pearson and Time lag correlations were also used to measure the correlation between those data.

**Results**: Moving Average analysis showed that Google Trends data have a linear time series pattern with official dengue report. Pearson correlation indicated high correlation for three defined search terms with R-value range from 0.921 to 0.937 (*p *≤ 0.05, overall period) which showed increasing trend in epidemic periods (2015–2016). Time lag correlation also indicated that Google Trends data can potentially be used for an early warning system and novel tool to monitor public reaction before the increase of dengue cases and during the outbreak.

**Conclusions**: Google Trends data have a linear time series pattern and statistically correlated with annual official dengue reports. Identification of information-seeking behavior is needed to support the use of Google Trends for disease surveillance in Indonesia.

## Background

Digital traces have become a potential data source for health-related purposes in the past few years. Digital epidemiology is a new field that uses digital traces to explore the patterns of disease and health dynamics in a population. The definition of digital epidemiology according to Salathe [1] is: ‘Digital epidemiology is epidemiology that uses data that was generated outside the public health system, i.e. with data that was not generated with the primary purpose of doing epidemiology.’

As the Internet penetration becomes more widespread, with increased mobile phone usage, and the growing artificial intelligence of machine learning, the field of digital epidemiology provides a promising approach to assist traditional surveillance systems [1,2]. This approach potentially fills the gap in conventional surveillance systems in developing countries that often suffer from underreporting, limited timeliness, and the lack of sufficient budget for physical needs, facilities, and infrastructures [3–6]. Data provided by conventional surveillance system often required weeks or months to be collected.

In Indonesia, regulation by Ministry of Health requested hospitals to report any new dengue cases to district health office within 24 hours after confirmed diagnosis [7]. However no single application was available to capture the data electronically. Consequently, each district has its own database structure of dengue cases. Data from districts are submitted monthly to province and national level. Reports at province and national level are aggregated on number of cases by districts, age group. Top-down feedbacks are provided by the sub-directorate of Vector-Borne Diseases and Zoonoses under the Directorate General of Disease Prevention and Control in the Ministry of Health of the Republic of Indonesia. This circumstances potentially caused the delay in response and indicated the need for an alternative data source to depict the dengue cases in near real-time.

Among the digital traces that are increasingly studied for epidemiology are those recorded in search engines [2]. These data provide the information-seeking patterns using specified search terms in defined locations during a specific time period. Digital recorded data provided by Google are displayed on Google Trends’ website (https://trends.google.com/trends/). Many studies proved that Google Trends data correlated well with traditional surveillance data [8–14]. Those researches reveal the potential use of Google Trends data that can be obtained earlier, more easily, and at little cost compared with conventional reporting systems. On the other hand, some studies reported a weak potential use of Google Trends data finding that they are more influenced by media clamor than truly actual epidemiological burden [15,16].

The increasing Internet penetration in Indonesia that has attained 54.7% and the huge utilization of Google (98%) indicate the potential use of Google Trends in Indonesia [17,18] This study was designed to validate the use of Google Trends data as an alternative or complement data source for dengue surveillance in Indonesia. No study was performed to measure the correlation between country-wide official dengue reports and Google Trends data in Indonesia. This is the first study to measure the correlation between Google Trends data on dengue fever and the Indonesian national surveillance report at a national level.

## Methods

This research was a quantitative study using time series data ranging from 2012 until 2016. The framework in this study is adapted from a previous study related to validation of Google Trends data at the national level [10]. We used official dengue reports from the Department of Arbovirus, Health Ministry, Indonesia and Google search volumes related to dengue in Indonesia from Google Trends. Official dengue reports were used as a gold standard to validate the Google Trends data.

Cases with confirmed status of dengue from laboratory tests that were reported in official dengue reports from 34 provinces in Indonesia are available on a monthly basis. Data cleaning was performed for those data to examine the completeness of data. Missing values from five provinces including Lampung, North Sulawesi, West Sulawesi, Papua, and West Papua are filled in using the Amelia Package in RStudio. This approach used multiple imputation and frequently used to overcome missing values in time series data [19]. Multiple imputation used a Bayesian approach to replace missing values with predictive distribution based on the observed data [20].

Complete official dengue reports then were transformed to the same interval of relative search volume (RSV) in Google Trends data, in order to compare the official dengue reports and Google Trends data in a single graphical form. This approach is also used in a previous study to transform the official dengue reports in interval data which range from 0 to 100 [10]. By using those approach, 0 is defined as the absence of dengue case and 100 is defined as the highest incidence of dengue cases during 2012 until 2016.

We compared the normalized dengue cases with dengue Google search volume for the same data period. Dengue Google search volume is described as how often a defined search term is used by Indonesians to search online information related to dengue in Google. Data were downloaded in comma-separated values (CSV) file from Google Trends’ website (https://trends.google.com/trends/) and are available on a weekly basis. Data were obtained using 19 search terms related to disease definition, symptom, treatment, and vector of disease which is listed in Table 1. Search terms were collected from Google Trends (search terms listing the most frequently used) and Google Correlate (search terms which have a similar pattern with the search term ‘*demam berdarah dengue*’).

**Table 1.** List of search terms.

Num	Category	Search Term	Description	Source
1.	Disease definition	‘*demam berdarah*’, ‘*dengue*’, ‘*dengue fever*’, ‘*fever*’, ‘*penyakit demam*’, ‘*penyakit demam berdarah*’	Terms are used to identify searching pattern related to disease definition in bahasa Indonesia	Google Correlate
		‘*demam berdarah dengue pdf*’, ‘*dengue hemorrhagic fever*’, ‘*dhf*’, ‘*demam berdarah dengue*’, ‘*dbd*’		Google Trends
2.	Symptom	‘*berdarah*’, ‘*demam*’	Terms are used to identify searching pattern related to dengue symptom in bahasa Indonesia	Google Correlate
		‘*gejala demam berdarah dengue*’, ‘*gejala demam berdarah*’		Google Trends
3.	Treatment	‘*obat demam berdarah dengue*’	Terms are used to identify searching pattern related to dengue treatment in bahasa Indonesia	Google Trends
4.	Vector of disease	‘*aedes*’, ‘*aedes aegypti*’, ‘*aegypti*’	Terms are used to identify searching pattern related to dengue vector in bahasa Indonesia	Google Correlate

Obtained data from Google Trends then were transformed from weekly period to monthly period using mean. This method was also used in previous study [11], in order to compare two sets of data in a single graphical form using a line chart in Microsoft Excel. Graphs which have relatively similar linearity of pattern then can be visualized using moving average analysis. Moving average analysis was used to measure the pattern similarity between official dengue reports and Google Trends data in more detailed ways. Pattern similarity includes the linearity of pattern, similarity of leap, and similarity of dengue outbreak per period in Indonesia.

Pearson correlation was performed for search terms with the highest pattern of similarity with official dengue reports. The correlation strength was defined as a correlation coefficient R-value of 0.7 (*p *≤ 0.05). We also performed Time lag correlation analysis with significance level at *p *≤ 0.05 for search terms with the highest correlation. Time lag correlation is used to compute the correlation between time lag variables and official dengue case history. Statistical analysis was conducted using Stata version 13.

## Results

**Figure 1.**
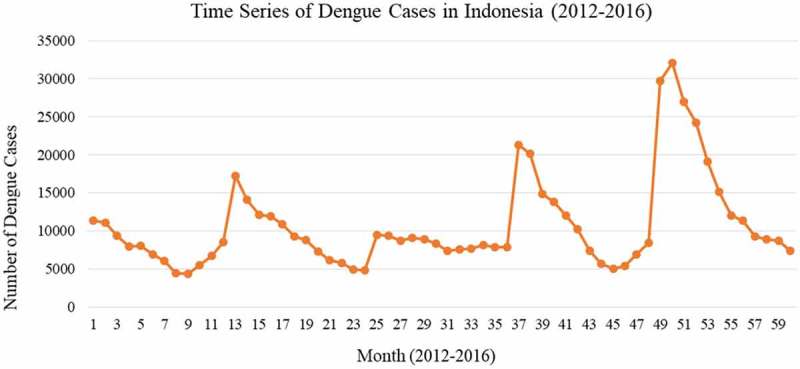
Time series of dengue cases in Indonesia (2012–2016).

Results of data analysis in Figure 1 show the time series of dengue cases in Indonesia from 2012 until 2016. There were four peaks of dengue cases with the highest peak in February 2016 which involved 32,117 dengue cases. Figure 1 shows the dengue outbreaks per period in Indonesia which tended to increase between October to January and then spiked to a peak in January or March.

**Figure 2.**
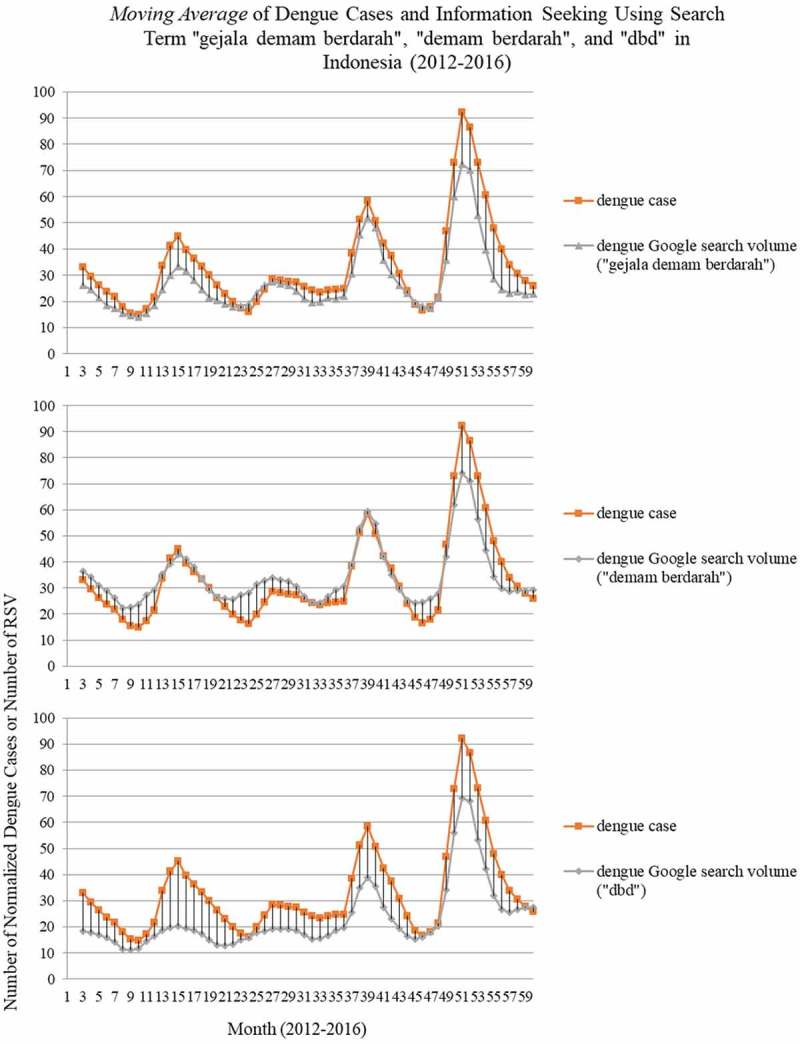
Moving average of dengue cases and information seeking using search term ‘gejala demam berdarah’, ‘demam berdarah’, and ‘dbd’ in Indonesia (2012–2016). DOI for Dataset: 10.17632/x855pphhx9.1

Time series of dengue cases then were visualized in single graphical form with Google Trends data. Moving average graph from official dengue reports and Google Trends data which has relative similarity in linearity of pattern is shown in Figure 2. Search terms such as ‘*gejala demam berdarah*’, ‘*demam berdarah*’, and ‘*dbd*’ seem to be in-line with official dengue reports. Information seeking using search term ‘*demam berdarah*’ increased in point 9 (22.6); 23 (27.2); 34 (26.8); and 46 (24.6). Search term ‘*dbd*’ increased in point 10 (11.6); 22 (13.4); 33 (15.5); and 46 (16.2), followed by search term ‘*gejala demam berdarah*’ which increased in point 11 (15.5); 23 (18.3); 33 (20); and 48 (21.4). Compared with official dengue reports which increased in point 11 (17.2); 25 (20); 34 (24.3); and 47 (18), search terms ‘*gejala demam berdarah*’, ‘*demam berdarah*’, and ‘*dbd*’ are increased in 1 until 3 points before the increase of dengue cases. There were 4 peaks in the last 5 years which are visualized by official dengue reports and the 3 search terms from Google Trends in point 15, 27, 39, and 51. Figure 2 also shows that information seeking using the search term, ‘*demam berdarah*’ tended to have higher value than official dengue reports, different from the search terms, ‘*gejala demam berdarah*’, and ‘*dbd*’ which tend to have lower values than official dengue reports.

Results of Pearson correlation in Table 2 show high correlation (R-value≥0.7 and *p *≤ 0.05) between official dengue reports and the Google Trends data. Correlations from the three search terms in the overall time period range from 0.921 to 0.937. The search term ‘*gejala demam berdarah*’ has the highest R-value in the overall time period. During the last 5 years, R-value seems to be increased in the epidemic period (2015–2016) and search terms ‘*gejala demam berdarah*’ and ‘*demam berdarah*’ seem to have stable R-value. Results of Time lag correlation in Table 3 show high correlation (R-value≥0.7 and *p *≤ 0.05) between official dengue reports and Google Trends data a month earlier which have R-value ranging from 0.755 to 0.773. Information seeking using the search term ‘*gejala demam berdarah*’ in the month prior shows the highest correlation with official dengue reports (R-value = 0.773; *p *≤ 0.05).

**Table 2.** Result of pearson correlation.

Time Period	Search Term		
	‘*gejala demam berdarah*’ (dengue symptom)	‘*demam berdarah*’ (dengue)	‘*dbd*’ (abbreviation of dengue)
Overall period	0.937*	0.931*	0.921*
2012	0.936*	0.918*	0.862*
2013	0.847*	0.850*	0.719*
2014	0.844*	0.814*	0.570
2015	0.921*	0.929*	0.918*
2016	0.954*	0.966*	0.950*

*significant in *p *≤ 0.05.

**Table 3.** Result of time lag correlation.

Time lag	Search Term					
	‘*gejala demam berdarah*’ (dengue symptom)		‘*demam berdarah*’ (dengue)		‘*dbd*’ (abbreviation of dengue)	
	r	*p*-value	r	p-value	r	*p*-value
−3	0.264*	0.047	0.283*	0.033	0.356*	0.007
−2	0.517*	<0.001	0.526*	<0.001	0.567*	<0.001
−1	0.773*	<0.001	0.755*	<0.001	0.767*	<0.001
0	0.937*	<0.001	0.931*	<0.001	0.921*	<0.001

*significant in p ≤ 0.05

## Discussion

Validation using moving average analysis showed that Google Trends data have a linear time series pattern correlated with official dengue reports. This finding is relevant to previous research by Cho [10]. Information seeking using the search term ‘*gejala demam berdarah*’, ‘*demam berdarah*’, and ‘*dbd*’ followed the dengue outbreak period in Indonesia from October to January and the peak in January to March during the epidemic years 2015 and 2016. Validation using Pearson correlation shows high correlation (R-value ≥0.7 and *p *≤ 0.05) between official dengue reports and Google Trends data. This finding is relevant to previous studies by Althouse, Chan, Cho, Gluskin, Castro, Strauss and Teng [8–14]. Those publications showed R-values ranging from 0.33 to 0.94. Researches in tropical countries [10,11,13,14] showed the high correlation (R-value ranging from 0.82 to 0.94) between official surveillance data with Google Trends data that seem to be similar with the result of this study. In comparison, the R-value in this research is relatively high (R-values ranging from 0.921 to 0.937).

The high correlation between official dengue reports and Google Trends data in this study is different from Alicino, Cervellin, and Ellery’s research finding [15,16,21]. Those researches found a disassociation between Google Trends data and disease occurrence and also found that Google Trends data is more influenced by media coverage than actual epidemiological burden. Thus, the potential use of Google Trends data depends on media coverage, Internet penetration, and utilization of mobile phone. Apart from those research findings, research by Chan found that information seeking related to dengue tended to be less influenced by media coverage [9].

According to the 2 steps of validation, moving average analysis and Pearson correlation, Google Trends data is well correlated with official dengue reports. This research successfully proved that Google Trends data is potentially useful as a complement data source for disease surveillance in Indonesia where Internet penetration attained 54.7% (2017). One previous study suggested that Google Trends is better suited in developed countries with large Internet penetration [22].

Three search terms with linear time series pattern and high correlation with official dengue case were drawn from disease definitions and symptom category. This research finding is relevant to research from Althouse, Chan, Cho, and Kang [8–10,23]. Search terms which are generally used by netizens have higher correlation with official data [10,23]. This finding also are demonstrated in this study. Search terms which generally are used by Indonesian netizens such as ‘*gejala demam berdarah*’, ‘*demam berdarah*’, and ‘*dbd*’ have higher correlation with official dengue reports than the search term ‘*demam berdarah dengue*’, even though that search term is a standard disease definition for dengue in Bahasa Indonesia. Different from the search term ‘ge*jala demam berdarah*’ and ‘*demam berdarah*’ that had generally stable R-values as shown in Table 2, the search term ‘*dbd*’ had a fluctuating R-value. The search terms ‘*gejala demam berdarah*’ and ‘*demam berdarah*’ are specific search terms that have specified result from query (‘*gejala demam berdarah’* has 104,000 results and ‘*demam berdarah*’ has 2,560,000 results, whereas the search term ‘*dbd*’ has a broad query with 20,400,000 results).

Different from previous research [8–10,23], search terms in this research were collected from Google Trends (search terms used most frequently) and Google Correlate (search terms which have a similar pattern with the search term ‘*demam berdarah dengue*’). According to the results of the Pearson correlation in Table 2, and list of search terms in Table 1, Google Trends and Google Correlate successfully describe the keyword or search term utilization by Indonesians. Nevertheless, the accuracy of keyword or search term identification depends on information-seeking behavior which are influenced by media trends, outbreak news briefs, disease occurrence, and Internet penetration [8,9,10,11,13,15,16,23]. Information-seeking behavior also is influenced by individual variables such as age, sex, level of education, cultural aspect, language, social class, marital status, level of healthcare utilization, and level of stress [23,25,26,27]. An additional factor that drives the information-seeking behavior is keyword suggestion in Google. According to Wen and Sun [24], keyword suggestion is generated from previous query using content-based re-ranking. Thus, keyword or search term utilization also depends on previous query. In summary, the condition, distribution, and dynamic of factors that influences the information-seeking behavior may vary among national wide and change over time. Therefore, identification of factors that influence the information-seeking behavior is needed to support the use of Google Trends for disease surveillance in Indonesia.

Increasing of information seeking in 1 until 3 points before the increase of dengue cases as shown in Figure 2 and high correlation of lag-1 in Time lag correlation indicate the initial potential use of Google Trends data as an early warning system. Some previous studies showed the potential use of Google Trends data as an early warning system in countries with a weak surveillance system [8,9,11,13,28]. This finding also indicates the potential use of Google Trends as novel tool to monitor public reaction before the increase of dengue cases and during the outbreak. Google Trends is potentially used to capture the public reaction in terms of worries, knowledge needs, and gaps which can be obtained earlier, more easily and at little cost [9,10,23,29].

With an assumption that information seeking related to dengue tended to be less influenced by media coverage [9], Google Trends can be potentially used to capture knowledge needs, and gaps between available information and needed information. Gaps can be identified using search term or keyword utilization by Indonesians in Google Trends and Google Correlate before the increase of dengue cases and during the outbreak. Identified gaps then can be used to determine the topic or information which is published on health official website and news channel. As the three most frequently accessed news channel in Indonesia, Tribunnews.com, Detik.com, and Liputan6.com could potentially be used to disseminate the needed information [30].

Early warning system according to World Health Organization is ‘timely surveillance systems that collect information on epidemic-prone diseases in order to trigger prompt public health interventions’ [31]. Approaches in utilization of Google Trends for early warning systems and as a monitoring tool for public reaction are intended to assist traditional surveillance systems in order to increase public health response to dengue in Indonesia. A study in Yogyakarta municipality reported that on average it takes 12 days to submit the report from hospital to the district health office [32]. Given that no standardized electronic dengue surveillance system, each district develop the system differently with limited interoperability. Consequently, provincial health offices and the Ministry of Health lacks of disaggregated data for appropriate action.

In this concern, Google Trends is a prospective tool to overcome the timeliness problem in conventional surveillance system which requires weeks or months to collect the data. In principle, this study offers opportunities to complement the existing surveillance system especially in terms of early warning. However, some works need to be done. These include overcome the noise to increase the quality of data, combine with other data source including social media and online news data, then create the algorithm to produce early warning systems. Google Trends also can be used to reveal what people search in a defined time and location. Otherwise, utilization of Google Trends as a monitoring tool for public reaction can be used immediately. Google Trends data can be used to monitor the public interest and the most commonly searched topic.

According to Bragazzi [33], Google Trends can be used to monitor the public interest and the most commonly searched topic, while Google Trends cannot be used to disclose the individual characteristics as done by conventional surveillance system. Therefore, Google Trends does not have the capacity to replace the existing surveillance systems [34] but can serve to supplement and complement them.

Implementation of Google Trends in Indonesia still poses some challenges related to Internet penetration and information-seeking behavior. As an island country, Indonesia has to encounter the discrepancy of infrastructure and level of literacy which may vary widely nationally. Those factors can affect the Internet utilization and information-seeking behavior in all regions in Indonesia. Google Trends could be used easily in a region with high Internet penetration and high dengue incidence. Nevertheless, how to implement the Google Trends in a region with high dengue incidence but low Internet penetration still remains challenging. Future studies need to validate the utilization of Google Trends data in regions with high dengue incidence and compare it among regions with high and low Internet penetration in Indonesia.

Googling for health or disease-related information does not always reflect the individual’s health condition. Likewise, searching for dengue information could also be performed by those with the infected disease in various point during the incubation period, other related disease with similar symptoms or even by healthy people [9]. Research by Indriani revealed the similarity of spatio-temporal patterns of dengue and chikungunya in Yogyakarta [35]. This issue poses a critical challenge for any Google Trends research. Thus, involving other variables that potentially influencing the googling behavior is needed to weigh the relative search volume and then improve the correlation analysis. Beside information-seeking behavior, other researchers could consider Internet penetration rate by geographic areas as a weighting variable for Google Trends data in order to increase the quality of data.

## Conclusion

Google Trends data have a linear time series pattern and are statistically correlated with official dengue report. Identification of information-seeking behavior is needed to support the use of Google Trends for disease surveillance in Indonesia.

## Paper context

This study has demonstrated that Google Trends data on dengue has a high potential to complement the national level surveillance data in Indonesia. In a dengue endemic country with 54.7% Internet penetration, Google Trends data could be adopted as an early warning system and monitoring tool of public responses to communicable diseases. Further validation studies at sub-national level are necessary since the district level surveillance system is highly influenced by the decentralization policy and interests of local leadership.
